# An Integrated Experimental/Theoretical Study of Structurally Related Poly-Thiophenes Used in Photovoltaic Systems

**DOI:** 10.3390/molecules21010110

**Published:** 2016-01-19

**Authors:** Davide Vanossi, Luigi Cigarini, Andrea Giaccherini, Enrico da Como, Claudio Fontanesi

**Affiliations:** 1 Department of Geological and Chemical Sciences, University of Modena and Reggio Emilia, Via G. Campi 183, Modena 41125, Italy; davide.vanossi@unimore.it; 2 Department of Physics, University of Modena and Reggio Emilia, Via G. Campi 213, Modena 41125, Italy; luigi.cigarini@unimore.it; 3 Department of Chemistry, University of Florence, Via della Lastruccia 3, Sesto Fiorentino 50019, Italy; andrea.giaccherini@unifi.it; 4 Department of Physics, University of Bath, Claverton Down Bath BA2 7AY, UK; E.Da.Como@bath.ac.uk; 5 Department of Engineering “Enzo Ferrari”, University of Modena and Reggio Emilia, Via Vivarelli 10, Modena 41125, Italy

**Keywords:** polythiophenes, band gap, DFT, HF, exciton

## Abstract

In this work, a series of eight thiophene-based polymers (exploited as “donors” in bulk heterojunction photovoltaics cells), whose structures were designed to be suitably tuned with the electronic characteristics of the [6,6]-Phenyl C61 butyric acid methyl ester (PCBM), is considered,. The electronic properties of the mono-, di-, trimeric oligomers are reckoned (at the Hartree-Fock and DFT level of the theory) and compared to experimental spectroscopic and electrochemical results. Indeed, electrochemical and spectroscopic results show a systematic difference whose physical nature is assessed and related to the exciton (electron-hole) binding energy ($J_{e,h}$). The critical comparison of the experimental and theoretical band gaps, *i.e.*, the HOMO-LUMO energy difference, suggests that electrochemical and DFT values are the most suited to being used in the design of a polythiophene-based p-n junction for photovoltaics.

## 1. Introduction

**Figure 1:**
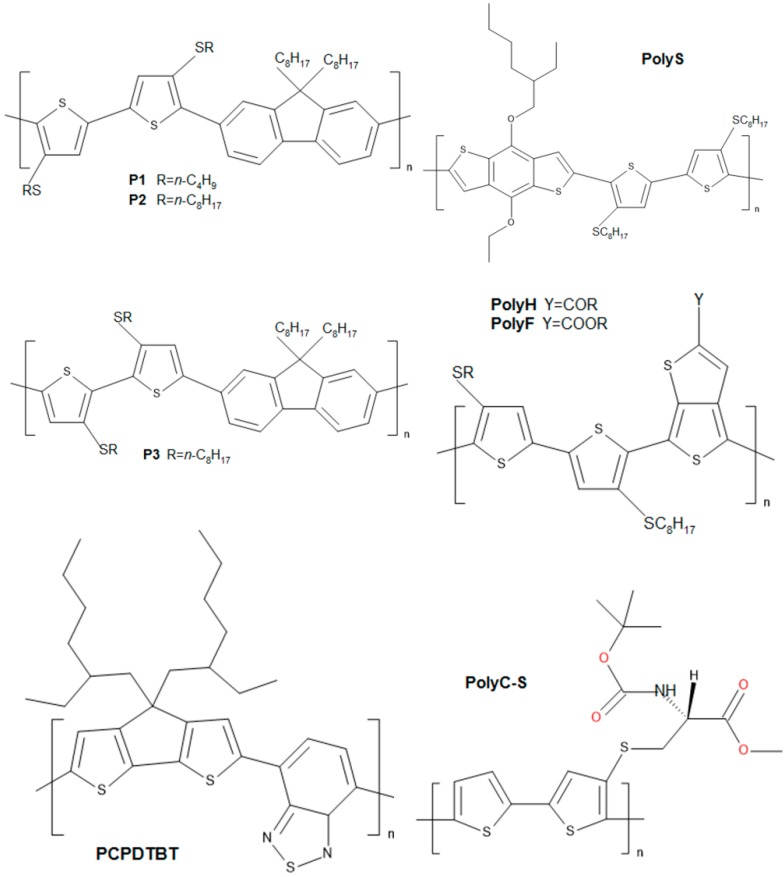
Polymer structures studied in this work.

The electronic properties of eight thiophene-based polymers (Figure 1 shows the relevant structures), with a particular focus on the interfacial behavior, are rationalized within a “Lego-like sum approach”: the electronic properties of the mono-, di-, trimeric species are calculated, and eventually the trimer results are selected (the differences between the dimer and trimer oligomers are almost negligible) and shown, in the following figures, to represent the electronic features of the polymer system well. Such a general modelistic approach spans extremely different worlds: from the Mott-Schottky barrier to Tafel plots in electrochemical systems [1,2,3]. It is noteworthy that when the field of linear conjugated polymers is considered, a number of non-linear effects concur to determine the final observed electronic properties (with a particular focus on conductivity), leaving this research topic still open to discussion and further development, because low dimensional structures such as polymers (polymers can be considered as electron one-dimensional conductive wires) are unstable, and, in these systems, the coupling between electrons and phonons wavefunctions (leading to the definition of the polaron [4,5]) determines a more tight localization of single- and double-bonds which lifts molecular orbitals’ degeneracy and finally induces the localization of π-electrons, leaving both the experimental and theoretical work a still-challenging field [4,5,6,7,8]. Within this field, the up-to-date frontier hot topic is the estimation and calculation of the so-called polaron dimension [9], which is thought to play a prominent role in determining the polymer electronic conduction.

Furthermore, a field of extremely relevant interest and expectations concerns thin-film polymer semiconductors exploited in hybrid systems, such as organic-based light-emitting diodes, photovoltaic cells and thin-film transistors, where the influence of the degree of order in the solid state plays a major role [10,11]. Indeed, the understanding of the interplay and relationship between the film morphology/electronic-structure and charge transport is of key importance for improving the performance of thin-film transistors [8].

In particular, in the field of organic semiconductors exploited to assemble photovoltaic devices, the open circuit potential is rationalized on the basis of the reciprocal HOMO-LUMO energy differences between the donor and the acceptor partners [11,12,13]. Although such an approach seems by far much too crude in its strategy, the straight comparison of HOMO-LUMO MOs energy levels, of the donor and acceptor building blocks, is still the most widespread tool exploited in the modelization of photovoltaic organic-based systems [14,15]. The electronic properties of the mono-, di-, trimeric oligomer species are considered and compared to the experimental spectroscopic and electrochemical results [16,17,18,19]. Among the different polythiophene structures investigated here, the peculiar characteristics of the chiral PolyC-S made it suitable for the realization of hybrid interfaces [20] exploited in the recently established field of “spin-dependent electrochemistry” [21,22].

## 2. Semiconductive Polythiophene Structures

In this work, a series of eight thiophene-based polymers (donors) are considered. Their structures were designed (both by the introduction of ring structures of various chemicals in the polymeric backbone structure—heteropolymers—and also by various lateral alkyl chains) to suitably tune the electronic properties of the PCBM (acceptor). The structures of the polymers studied here, are shown in Figure 1. PCPDTBT was purchased by Sigma-Aldrich (St. Louis, MO, USA) (754005 Aldrich, CAS Number 920515-34-0), while all of the other compounds are of original synthesis [16,17,18,19].

## 3. Experimental Setup

**Figure 2:**
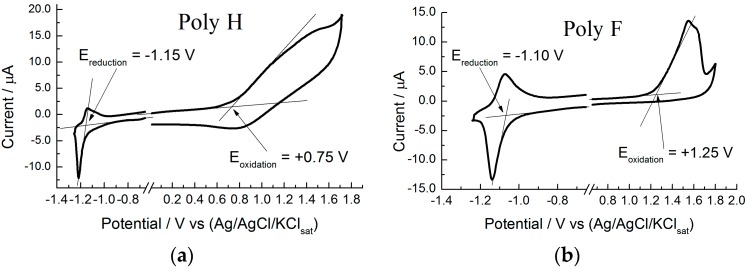
Cyclic voltametries (**a**,**b**) of the Poly H and Poly F polymer films on GC surfaces (obtained by drop-casting procedure), respectively. Showing the method adopted to determine the reduction and oxidation onset potentials, which are used to calculate the so-called electrochemical band gap ($\Delta E_{EC}$). Onset values are obtained by the intercept of the lines interpolating the baseline and redox peak currents.

UV-vis spectra were recorded in ambient air at room temperature (25 °C), by means of a Perkin Elmer Lambda 900 spectrophotometer (Perkin Elmer, Waltham, MA, USA). Cyclic voltammetry (CV) measurements were performed both with a CHI 660A Electrochemical Workstation (CH Instruments, Inc., Austin, TX, USA) and an AUTOLAB PGSTAT20 (Metrohm Autolab B.V., Utrecht, The Netherlands). A three-electrode electrochemical cell configuration was adopted. A 0.1 mol/L *n*-tetrabutyl ammonium hexa fluorophosphate (TBAPF_6_ > 99.9%, Sigma-Aldrich Chemie B.V., Zwijndrecht, The Netherlands) in acetonitrile (ACN) solution was used as the base electrolyte. The working electrode is obtained by drop-casting of polythiophene/CH_2_Cl_2_ solution applied on freshly polished Glassy Carbon (GC) electrodes (Metrohm Schweiz AG, Zofingen, Switzerland) and HTW Sigradur (HTW Hochtemperatur-Werkstoffe GmbH, Thierhaupten, Germany). Prior to polymer drop-casting, the GC surface was mechanically polished with emery paper, then with 0.05 μm alumina (Buheler, Lake Bluff, IL, USA), finally followed by a 5 min sonication cleaning in water. The GC surface was polymer-coated by casting one drop of a 0.1 mg/mL, in CH_2_Cl_2_, polymer solution on top of the GC surface and allowing it to dry [16]. A platinum wire was used as the counter electrode. A silver wire was used as a quasi-reference electrode, whose stability was checked (at the end of each measurement session) with respect to the ferrocene/ferrocenium reversible redox couple. In the following all potential values are referred to the Ag/Ag^+^ couple. A large number of screening experiments were carried out, varying both the dose of the drop-casting as well as the drying time. Films obtained on repeating the sequence of a single drop-cast application three times, followed by 15 min drying, allowed for the best CV reproducibility. We estimate that our reduction and oxidation potentials are affected by a 30 mV absolute error (±15 mV error). The electrochemical cell was de-aerated with argon for 15 min before any measurement session. Figure 2 shows two examples of the cyclic voltammetry data treatment, in order to show in detail how the electrochemical onset potentials were determined. Two completely different experimental behaviors are considered: Poly H characterized by a rather hill-defined/sluggish cyclic voltammetry pattern (with particular reference to the positive potential range: polymer film oxidation), Figure 2a, and Poly F which shows a rather well-defined and neat cyclic voltammetry pattern, Figure 2b.

**Figure 3:**
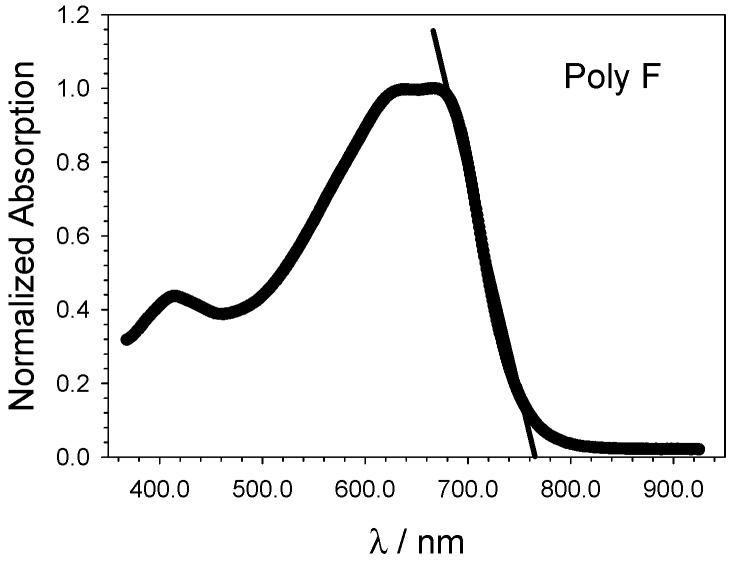
Poly F UV-vis spectrum, the line used to determine the onset wavelength is shown. The intercept with the absissa allows us to determine the optical band gap ($\Delta E_{OPT}$).

Figure 3 shows, as an example, the treatment of the UV-vis spectrum of Poly F, in order to show in detail how the onset wavelength is determined, which eventually leads to the calculations of the $\Delta E_{OPT}$ value.

## 4. Computational Details

In the present work, the overall calculations were performed in the framework of *ab initio* methods using the Gaussian and Firefly [23] QC packages, which are partially based on the GAMESS (US) source code. All calculations, unless otherwise indicated, were performed using C_1_ symmetry and are of restricted nature. The results presented in this paper are obtained both at the very basic Hartree-Fock (HF) and Becke, three-parameter, Lee-Yang-Parr (B3LYP) exchange-correlation density functional levels of the theory; the all-electron split valence plus polarization basis set 6-31G(d) was used in both HF and DFT calculations. Preliminary screening calculations were carried out using less accurate basis sets: LanL2DZ, 3-21G*, with focus on the influence of the geometry optimization as well as of the number of repetitive units in the oligomer on the variation of the HOMO-LUMO energies and band gap. Moreover, periodic boundary condition (PBC) calculations were performed, the latter results well compare with the HOMO-LUMO band gap relevant to the dimeric and trimeric species, together with the systematic calculation (again involving the mono-, di-, trimer species) of TDDFT electronic spectra [24].

## 5. Theoretical Background

### 5.1. Orbital Energies: DFT vs. HF

It is well established [25] that the Kohn-Sham orbitals, $\varphi_{i}\left(r\right)$, do not have any particular physical meaning; they only serve as a tool to construct the exact (at least in principle) ground-state density for the actual system of interacting electrons. The same consideration is clearly also directed for the Kohn-Sham eigenvalues, $\varepsilon_{i}$, which, from a formal point of view, are simply lagrangian multipliers inserted “*ad hoc*” to perform the constrained variational minimization. Nevertheless, there is one important exception to this statement when the energy $\varepsilon_{N}$ of the highest occupied orbital, for a finite system of *N* electrons, is considered: minus $\varepsilon_{N}$ depicts, in fact (at least in the case of an exact exchange-correlation functional [25,26]), the ionization potential $I_{p}\left(N\right)$ of the system, *i.e.*,:

(1) $$\varepsilon_{N}\left(N\right)=E\left(N\right)-E\left(N-1\right)=-I_{p}\left(N\right)$$

where E(N) and E(N−1) are the total ground-state energies for the system with *N* and *N −* 1 electrons. On the contrary, all the Hartree-Fock mono-electronic energies, thanks to the Koopmas theorem [27], correspond to electron removal energies once correlation and orbital relaxation effects are neglected. It is also possible to write down an exact and equivalent relation to Equation (1) starting from the Kohn-Sham energy $\varepsilon_{N+1}$ of the highest occupied orbital for a system with *N* + 1 electrons:

(2) $$\varepsilon_{N+1}\left(N+1\right)=E\left(N+1\right)-E\left(N\right)=-I_{a}\left(N\right)$$

Equations (1) and (2) can be considered the DFT Koopmans theorem, which, however, in comparison to the Hartree-Fock version, is exact because $\varepsilon_{N}$ is endowed with a many-bodies nature. Equation (2) shows that minus $\varepsilon_{N+1}$ is equal to the electron affinity $I_{a}\left(N\right)$ for the *N*-electron system (being E(N+1), the total ground-state energy for the system with *N* + 1 electrons).

Despite the soundness of Equations (1) and (2), differences in Kohn-Sham energy eigenvalues do not correspond, in general, to the exact excitation energies for the interacting *N*-electron system but, sometimes, they can be used as first, rather fairly acceptable approximations [28].

### 5.2. Computing the Band Gaps

When different types and states of matter are considered, it is well known [26,28,29] that energy band gaps may or may not occur. A gap is the direct manifestation of the existence of a finite energetic difference between two states of the system. In physics there are several type of gaps: the particle (or quasi-particle) gap, optical gap, superconducting gap [28]. For each one of these gaps, different experimental characterization methods exist. For example, the optical gap, which is related to the energy difference between the electronic ground state and the first excited state for a system with a fixed particle number *N* (neutral excitation), can be addressed by means of optical spectroscopy. Otherwise, the particle gap (which is of fundamental importance in an insulating periodic solid) is related to the ground-state energies of systems with different particle numbers (charged excitation) and can be typically probed using photoelectron spectroscopy. The particle gap is defined as [26,28]:

(3) $$E_{g}\left(N\right)=I_{p}\left(N\right)-I_{a}\left(N\right)$$

In terms of KS energies, Equations (1) and (2) can be rearranged as:

(4) $$E_{g}\left(N\right)=\varepsilon_{N+1}\left(N+1\right)-\varepsilon_{N}\left(N\right)$$

Equation (4) involves the energies of the highest occupied orbitals of two systems with a different number of electrons, so we are dealing with an excitation which does not conserve the number of electrons. Equation (4) can be clearly used when a finite system (such as a molecule) is considered, but becomes quite impractical when referring to a solid. In this last situation it is necessary to define a different particle gap, also known as a Kohn-Sham gap, by means of the following equation:

(5) $$E_{g}^{KS}\left(N\right)=\;\varepsilon_{N+1}\left(N\right)-\varepsilon_{N}\left(N\right)\equiv \Delta E_{DFT}$$

in which the lowest unoccupied (LUMO) and the highest occupied (HOMO) orbital energies, for the system with *N* electrons (neutral excitation), are considered. It is worthwhile to note that a relation formally equivalent to Equation (5) is commonly also adopted in the Hartree-Fock framework (even for finite systems):

(6) $$E_{g}^{HF}\left(N\right)=\varepsilon_{N+1}^{HF}\left(N\right)-\varepsilon_{N}^{HF}\left(N\right)\equiv \Delta E_{HF}$$

Equations (4) and (5) can be related to one another in this way:

(7) $$E_{g}\left(N\right)=E_{g}^{KS}\left(N\right)+\Delta_{xc}$$

where $\Delta_{xc}$, the many-bodies correction to the Kohn-Sham particle gap, can be casted as [28]:

(8) $$\Delta_{xc}=\varepsilon_{N+1}\left(N+1\right)-\varepsilon_{N+1}\left(N\right)$$

### 5.3. Optical and Electrochemical Band Gaps

**Figure 4:**
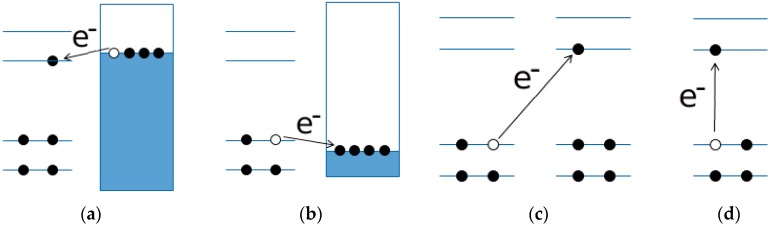
The white circles represent holes and the black circles electrons. In (**a**,**b**) the pseudo-fermi level of the polarized electrode is represented by the line on top of the dark rectangle representing the conduction band of the solid; (**c**,**d**) depicts the intermolecular and intramolecular electronic transitions, respectively. The other lines represent the orbitals in the polymer molecules while the arrows represent the electron transfer.

Absorption of a photon with energy equal to or greater than the band gap results in the excitation of an electron from the valence to the conduction band, leaving a hole in the valence band. Such an electron hole pair, or exciton, is bound by the electrostatic attraction between the collective state’s electron in the conductive band and the hole in valence band. By means of optical excitation, four basic types of charge-transfer processes are proposed by Credi *et al*. [30], and we proposed them also for semiconducting polymers: electron injection into a neutral molecule (Figure 4a), electron extraction from a neutral molecule (or hole injection, Figure 4b), removal of an electron from one molecule and placing it into an identical molecule at a infinite distance, (Figure 4c), and generation of an electron-hole pair within the same molecule (Figure 4d).

The electrochemically determined band gap ($\Delta E_{EC}$) is operatively defined as the difference in energy between the first reduction and the first oxidation processes of the molecule, respectively, corresponding to the process in Figure 4a,b. Thus is equivalent to the energy required to produce a noninteracting electron-hole pair (quasi-particle gap). The optical band gap $\Delta E_{OPT}$ corresponds to the process in Figure 4d. In the ground of this theoretical model, the two band gaps are related by Equation (9):

(9) $$\Delta E_{OPT}=\Delta E_{EC}+J_{e,h}$$

where $J_{e,h}$ is the binding energy of the exciton. Hence, for any given semiconducting molecule, the electrochemical energy gap is expected to be larger than the optical energy gap.

## 6. Results

**Figure 5:**
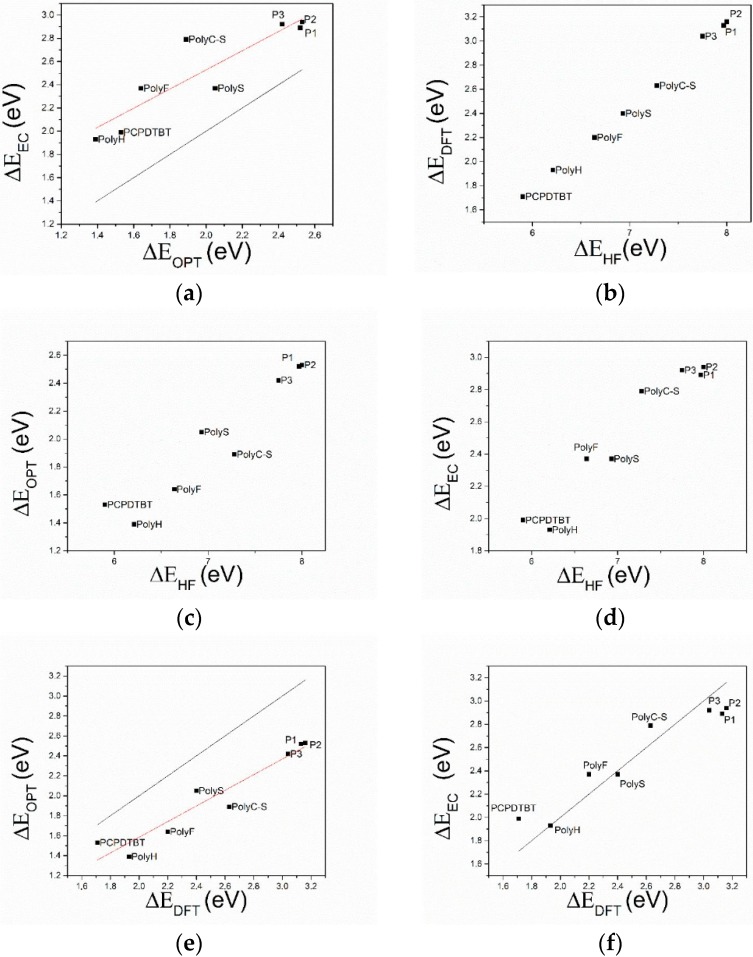
Comparison between (**a**) electrochemically determined and spectroscopically determined band gap; (**b**) band gap computed by means of DFT and HF methods; (**c**) band gap spectroscopically determined and computed by means of HF method; (**d**) band gap electrochemically determined and computed by means of HF method; (**e**) band gap spectroscopically determined and computed by means of DFT method; (**f**) band gap electrochemically determined and computed by means of DFT method. All data are reported in eV. The “equivalence line” shows the ideal line featuring slope = 1 and intercept = 0.

Figure 5a shows the $\Delta E_{EC}$ *vs.* $\Delta E_{OPT}$ graph, obtained by the experimental band gap values. The latter are obtained (i) by means of spectroscopical measurements, $\Delta E_{OPT}$ (optical band gap, calculated by the onset of the absorption peak determined from the UV/Vis spectra), and (ii) by means of CV curves, ΔE_EC_ (note that the difference in the onset of potentials, V, relevant to the reduction and oxidation current peaks is straight transformed in a band gap energy, eV, on the basis of the work of Trasatti [31]).

**Figure 6:**
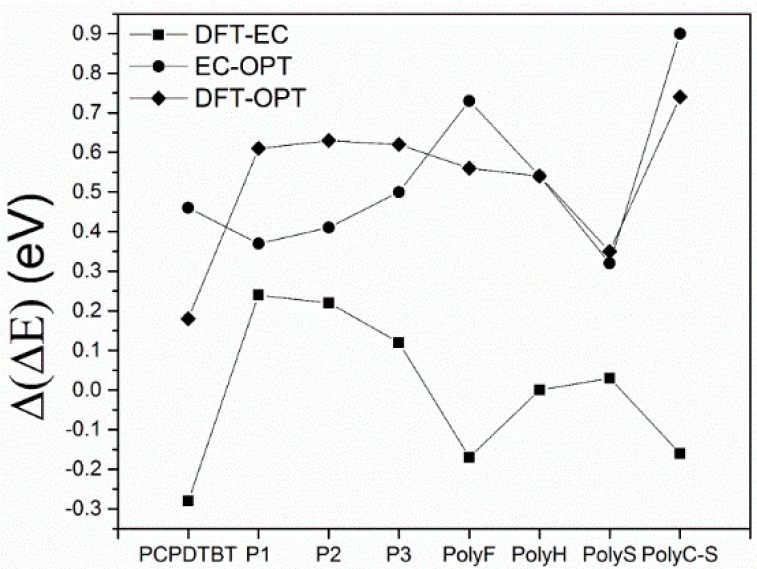
Discrepancies between band gaps computed by means of DFT method and electrochemically determined; band gaps electrochemically and spectroscopically determined; band gaps computed by means of DFT method and spectroscopically determined.

All the points in this dataset are more positive than the “equivalence line”: the electrochemical band gap is systematically larger than the optical band gap (about 0.45 eV), suggesting a bias in the difference between the two band gaps. In Figure 5b, the LUMO/HOMO energy difference, the HF band gap ($\Delta E_{HF}$) has been compared with the DFT band gap ($\Delta E_{DFT}$). Note the systematic large difference between ΔE_HF_ and ΔE_DFT_ values. Figure 5c,e patterns demonstrate that ΔE_HF_ are overestimated with respect to both the optical and electrochemical band gap, while in Figure 5d a comparison between the DFT band gap and the optical band gap shows that DFT systematically overestimates the optical band gap. Eventually, the electrochemical band gap is compared with the DFT gap (Figure 5f), and ΔE_DFT_ values semi-quantitatively match the electrochemical band gap: the equivalence line is almost exactly placed in the middle of the dataset. Figure 6 shows the difference between the DFT, electrochemical and optical band gap values (*i.e.*, ($\Delta E_{DFT}-\Delta E_{EC}$), ($\Delta E_{EC}-\Delta E_{OPT}$) and ($\Delta E_{DFT}-\Delta E_{OPT}$) differences). Notably, the difference between electrochemical and DFT band gaps ($\Delta E_{DFT}-\Delta E_{EC}$) is scattered homogenously around zero, and the maximum deviation is 0.28 eV. However, the discrepancy between electrochemical and optical results is the same as the DFT and optical one: both ranging between 0.2 and 0.9 eV, highlighting that the DFT and electrochemical band gaps have the same quantitative trend.

## 7. Conclusions

The electronic characteristics, with a focus on the band gap, of eight thiophene-based semiconducting polymers have been determined both experimentally (electrochemically, spectroscopically) and theoretically (at the Hartree/Fock and DFT level of the theory). Careful comparison between theoretical and experimental results allows us to draw some main line of action that is useful when dealing with problems of reciprocal coupling of hybrid donor/acceptor systems, where band gap features are thought to determine the ultimate performances of a system/device.

(1) The comparison of HF and DFT theoretical data, with both electrochemical and spectroscopic experimental band gap values, shows that the HF approach provides a dramatic overestimation of the band gap. The exchange-correlation and electron-correlation cannot be neglected; they have to be taken into account to assess the correct band gap energy. Indeed, ΔE_DFT_ values definitively show a better quantitative match with both the electrochemical and spectroscopic band gap values, as it is shown in Figure 5 and Figure 6.
(2) Arguments, based both on the purely modelistic (Figure 4) and on the comparison between DFT and experimental data (Figure 5 and Figure 6), show that the most effective approach to be used when assessing the band gap characteristics for photovoltaic materials is to make a reference to both the DFT and electrochemical methods to determine the HOMO-LUMO band gap.
(3) Eventually, an empirical quantitative value can be determined for the exciton stabilization energy ($J_{e,h}$), *vide infra* relation 8. The close comparison of the physics underlying absorption in electronic spectra (Figure 4d) and reduction/oxidation current peaks in cyclic voltammetry measurements (Figure 4a,b) together with the systematic difference observed in Figure 5a (the least square fit yields ΔE_EC_ = 0.53 + 0.99 ΔE_OPT_) allow us to propose a value of about 0.5 eV (the intercept of the least square fit) as an average value for the exciton stabilization energy [30,32]. Such an estimate is further supported by the systematic shift observed in Figure 6 between the ΔE_OPT_ *vs.* ΔE_DFT_ pattern (red line represents the least square fitting of the ΔE_OPT_ *vs.* ΔE_DFT_ data) and the relevant equivalence line.

As a whole, the results presented in this paper strongly support the view of a tight equivalence between the DFT and electrochemically determined band gap values (a result consistent both in terms of the physics underlying the different processes involving electrons and of the purely measured and computed values). The electrochemical measurements, giving an insight in the relative energy of HOMO and LUMO in different systems, seem to provide the values best suited for designing and selecting optimum candidates for organic/hybrid photovoltaic systems.
